# Survival and Inactivation by Advanced Oxidative Process of Foodborne Viruses in Model Low-Moisture Foods

**DOI:** 10.1007/s12560-020-09457-7

**Published:** 2021-01-27

**Authors:** Neda Nasheri, Jennifer Harlow, Angela Chen, Nathalie Corneau, Sabah Bidawid

**Affiliations:** 1 National Food Virology Reference Centre, Bureau of Microbial Hazards, Food Directorate, Health Canada 251 Sir Frederick Banting Driveway, Ottawa, ON K1A 0K9 Canada; 2 Department of Biochemistry, Microbiology and Immunology, Faculty of Medicine, University of Ottawa, Ottawa, ON Canada

**Keywords:** Hepatitis A virus, Norovirus surrogates, Low-moisture foods, Bead-based assay, Advanced oxidative process

## Abstract

Enteric viruses, such as human norovirus (NoV) and hepatitis A virus (HAV), are the major causes of foodborne illnesses worldwide. These viruses have low infectious dose, and may remain infectious for weeks in the environment and food. Limited information is available regarding viral survival and transmission in low-moisture foods (LMF). LMFs are generally considered as ready-to-eat products, which undergo no or minimal pathogen reduction steps. However, numerous foodborne viral outbreaks associated with LMFs have been reported in recent years. The objective of this study was to examine the survival of foodborne viruses in LMFs during 4-week storage at ambient temperature and to evaluate the efficacy of advanced oxidative process (AOP) treatment in the inactivation of these viruses. For this purpose, select LMFs such as pistachios, chocolate, and cereal were inoculated with HAV and the norovirus surrogates, murine norovirus (MNV) and feline calicivirus (FCV), then viral survival on these food matrices was measured over a four-week incubation at ambient temperature, by both plaque assay and droplet-digital RT-PCR (ddRT-PCR) using the modified ISO-15216 method as well as the magnetic bead assay for viral recovery. We observed an approximately 0.5 log reduction in viral genome copies, and 1 log reduction in viral infectivity for all three tested viruses following storage of select inoculated LMFs for 4 weeks. Therefore, the present study shows that the examined foodborne viruses can persist for a long time in LMFs. Next, we examined the inactivation efficacy of AOP treatment, which combines UV-C, ozone, and hydrogen peroxide vapor, and observed that while approximately 100% (4 log) inactivation can be achieved for FCV, and MNV in chocolate, the inactivation efficiency diminishes to approximately 90% (1 log) in pistachios and 70% (< 1 log) in cereal. AOP treatment could therefore be a good candidate for risk reduction of foodborne viruses from certain LMFs depending on the food matrix and surface of treatment.

**Supplementary Information:**

The online version contains supplementary material available at 10.1007/s12560-020-09457-7.

## Introduction

Gastroenteritis outbreaks and illnesses due to the consumption of contaminated low-moisture foods (LMFs) are recently emerging as a food safety burden in developed countries, with norovirus and hepatitis A virus being important causes of LMF-associated outbreaks (Carvalho et al. 2012; Donnan et al. 2012; Anonymous 2019; Park et al. 2015; Sakon et al. 2018). For example, a norovirus outbreak in 2008, which led to 117 symptomatic infections in Korea was attributed to dried radish (Yu et al. 2010), another norovirus outbreak in 2017, which involved 2094 cases in Japan was linked to dried seaweed (Sakon et al. 2018), and sun-dried tomatoes were identified as the vehicle for a large hepatitis A outbreak in Australia (Donnan et al. 2012).

Due to their low water activity (*a*_w_), LMFs are considered less susceptible to the growth of foodborne pathogens (Sanchez-Maldonado et al. 2018). For this reason, minimal intervention strategies are implemented during the production of LMFs (Sanchez-Maldonado et al. 2018). However, foodborne viruses are relatively resistant to various environmental factors (e.g., pH, temperature, and *a*_w_), and viral persistence in various environments, such as food surfaces and soil, has been demonstrated (Rzezutka and Cook 2004; Nasheri et al. 2019; Yeargin and Gibson 2018). Following the contamination of food and food surfaces with foodborne viruses through different mechanisms such as infected food-handlers, contamination with sewage (Shukla et al. 2018), the virus can remain infectious for a relatively long time (Cook et al. 2018, 2016). The environmental stability of foodborne viruses together with their low infectious dose and resistance to conventional antimicrobial treatments, could lead to their persistence in LMFs and cause serious implications on public health.

Little is known about the survival and inactivation of viruses in LMFs. In our previous study, we have developed a magnetic bead-based assay for the isolation of HAV and norovirus surrogates, feline calicivirus (FCV) and murine norovirus (MNV), from LMFs and compared its efficacy against the adapted ISO 15216–1 method (Nasheri et al. 2020). We have demonstrated that while both methods can be employed to recover viruses from LMFs, each method has its own advantages and disadvantages (Nasheri et al. 2020; Suresh et al. 2019). Thus, both methods were applied in the present study to investigate viral persistence and infectivity during 4-week storage in LMFs. Three popular LMFs that are usually consumed without further processing (i.e. chocolate, pistachios, and cereals), were selected for this study. Although, at present, no outbreaks or confirmed cases of foodborne viral disease have been associated with the consumption of these LMFs, they have been previously implicated in outbreaks associated with *Salmonella* (Hasani et al. 2020; Santillana Farakos et al. 2014), and voluntary recalls have been made due to suspected contamination with *Listeria monocytogenes* (Ly et al. 2020). Viral survival on each matrix was examined by plaque assay as well as droplet-digital RT-PCR (ddRT-PCR).

It is important to find an effective tool to mitigate enteric viruses from ready-to-eat LMFs, therefore, we explored the efficiency of advanced oxidative process (AOP), which combines UV-C radiation with ozone and hydrogen peroxide vapor treatments for the inactivation of pathogens in ready-to-eat foods. UV-C treatment has been used with relative success to eliminate foodborne viruses in fresh produce and food-contact surfaces (reviewed in (Cook et al. 2018; Bosch et al. 2018)). The virucidal efficacy of ozone against foodborne viruses is variable and depends on several factors including the matrix and the ozone concentration (dose) (Yeargin et al. 2016). It is believed that ozone treatment affects the viral capsid and genome, however, its inactivation mechanism is poorly understood (Brie et al. 2018). Hydrogen peroxide (H_2_O_2_) acts as an oxidant against bacteria and viruses by producing hydroxyl-free radicals, which affect viral capsid and genome. Moreover, H_2_O_2_ breaks down to water and oxygen and thus constitutes a low environmental risk (Zonta et al. 2016; Becker et al. 2019b). The efficacy of H_2_O_2_ treatment against HAV and surrogates of norovirus has been shown to be matrix-dependent [reviewed in (Cook et al. 2018; Bosch et al. 2018)]. In this study, we evaluated the combinational effect of these inactivation strategies against foodborne viruses in select LMFs by cell culture based infectivity assays as well as ddRT-PCR assay.

## Materials and Methods

### Cells and Viruses

Crandell Rees feline kidney (CrFK) cells were obtained from ATCC (# CCL-94) and maintained as previously described (Bidawid et al. 2003). Feline Calicivirus (FCV) (ATCC # VR-782) was used to inoculate LMFs.

Murine BV-2 cells (kindly provided by Dr. Wobus, University of Michigan, Ann Arbor) were grown in Dulbecco’s modified Eagle’s medium (Invitrogen, Burlington, Ontario, Canada) supplemented with 10% fetal bovine serum, 0.1 mg/mL l-glutamine, and 0.1 mg penicillin–streptomycin. The cells were incubated at 37 °C with 5% CO_2_ and maintained as previously described (Cox et al. 2009)**.** MNV (kindly provided by Dr. Virgin, Washington University School of Medicine, St. Louis, MO) was used to inoculate LMFs.

Hepatitis A virus strain HM-175 (ATCC # VR-1402) and seed cultures of fetal rhesus monkey kidney cells (FRhK-4) were kindly provided by Dr. S. A. Sattar (University of Ottawa). FRhK-4 cells were grown, maintained and prepared as described previously (Mbithi et al. 1991).

### Plaque Assay

Virus titres were quantified by plaque assay in 12-well plates (Millipore Sigma, Cat# Z707775). For the quantification of FCV, MNV, and HAV, the cell lines CrFK, BV-2, and FRhk-4, respectively, were infected with the viruses extracted from different low-moisture foods. The cell monolayers were grown at 37 °C overnight, and each of three wells was inoculated with 100 µL of the sample dilutions from virus suspension extracted from each LMFs (pistachios, chocolate or cornflakes). Virus suspensions were diluted using 1 × Earle's Balanced Salt Solution (EBSS) (Thermofisher, Cat# 24010-043), and were adsorbed for 60 min at 37 °C, with gentle rocking. A 2 mL mixture of agarose-media was overlaid onto monolayers and the plates were incubated at 37 °C with 5% CO_2_ for 2 days (FCV and MNV) or 8 days (HAV). The monolayers were fixed with 3.7% paraformaldehyde (Millipore Sigma, Cat# F1635) for a minimum of 4 h. The monolayer was stained with 0.1% crystal violet and plaques were counted manually and converted to PFU/mL.

### Inoculation

Chocolate liqueur (Cargill) was weighed in 4 g amounts into wells of a 6-well plate. Chocolate was melted at 43 °C in a bead bath, solidified and was stored at room temperature until samples were ready for virus inoculation. Chocolate samples in each well were spot inoculated with 100 µL of 5 × 10^6^ PFU/mL of FCV (6.2 × 10^6^ genome copies/µL), 4 × 10^6^ PFU/mL of MNV (1.05 × 10^6^ genome copies/µL), or 1 × 10^5^ PFU/mL of HAV (1.2 × 10^5^ genome copies/µL) by pipetting small volumes to ensure even distribution of the virus. The inoculated chocolate was dried at room temperature (RT) in a biosafety level 2 cabinet (BSC) for 1 h. Samples were inoculated in triplicate (3 wells of 4 g of chocolate per time point) in two sets. The samples at *T*_0_ were analyzed immediately and the rest of the samples were incubated at RT for 24 h, 1 week, 2 weeks, 3 weeks and 4 weeks for survival assay prior to extraction.

Twenty-five grams of unsalted dry-roasted, shelled pistachios from American Pistachio Growers (APG) were weighed in petri dishes in two sets, each in triplicate. The pistachio samples were inoculated as described before for chocolate.

Cornflakes (Kellogg’s) were weighed in 25 g amounts in petri dishes in two sets, each in triplicate, and were inoculated as described before.

The relative humidity (RH) of our lab for the duration of the survival studies ranged between 25 and 28%. The initial a_w_ values for uninoculated chocolate liqueur, cornflakes and pistachios were 0.03 ± 0.00, 0.30 ± 0.00 and 0.14 ± 0.05. The inoculated chocolate liquor, cornflakes and pistachios were dried to approximately pre-inoculation levels, i.e., 0.13 ± 0.02, 0.23 ± 0.03 and 0.19 ± 0.05, respectively.

### Recovery Using ISO 15216-1:2017 Method (ISO. 2017)

To recover FCV, MNV, or HAV, from the inoculated chocolate, the entire surface of the chocolate was swabbed five times with a sterile cotton swab pre-moistened with 1 × phosphate-buffered saline (PBS, pH 7.2). The swab was then immersed in 500 µL PBS for virus quantification by plaque assay or lysis (AVL) buffer for viral RNA extraction using Qiagen’s QIAmp Viral RNA mini kit according to manufacturer’s instructions.

To recover viruses from inoculated pistachios and cornflakes, the 25 g of samples were added to mesh filter bags (VWR, #11216-904, PA) and 40 mL of TBGE buffer (100 mM Tris pH 9.5, 50 mM glycine, 1% wt/vol beef extract) was added to each sample. Next, 175 mL of TGBE buffer was added and the sample was mixed and incubated at room temperature, on a rocking plate for 20 min. The eluate was transferred to a 50 mL centrifuge tube and centrifuged at 10,000×*g* for 30 min at 4 °C. The supernatant was decanted into a new tube and the pH was adjusted to 7 ± 0.5 with HCl. 5 × polyethylene glycol 6000 (500 g/L PEG, Sigma)/NaCl (1.5 mol/L Fisher) of 1/4 volumes of the weight of each sample was added to each tube, followed by incubation on ice, on a rocking plate for 1 h. Samples were centrifuged at 10,000×*g* for 30 min at 4 °C. The supernatant was discarded and the pellet was resuspended in 500 µL of PBS for pistachios and 1000–2000 µL PBS depending on the size of the pellet for cornflakes, then stored at − 80 °C for further analysis by plaque assay or ddRT-PCR.

Following viral recovery from inoculated LMFs, viral RNA extraction was performed using QIAmp Viral RNA mini kit according to manufacturer’s instructions. The recovery efficiency for each LMF is calculated as described before (Nasheri et al. 2020) and is shown in Supplementary Tables 1 and 2.

### Recovery Using Bead-Based Method

A 1:10 bead to sample ratio was used for all the bead-based extractions (100 µL of beads for 1 mL of eluate)**.** Porcine gastric-coated MagnaBind carboxyl derivatized magnetic beads (Thermofisher) (PGM-MB) were prepared as described previously (Suresh et al. 2019; Dancho et al. 2012). HAV-Ab coupled beads were prepared using the magnetic DynaBeads Antibody Coupling Kit (Thermofisher, Cat# 14311D) according to manufacturer’s instructions. To recover virus from inoculated chocolate, the surface was swabbed with a pre-moistened cotton swab five times and the virus was released in 800 µL PBS and extracted using 80 µL PGM-MB beads.

To recover virus from inoculated pistachios and cornflakes, the samples were placed in mesh filter bags with 40 mL of PBS for pistachios, and 175 mL of PBS for cornflakes. The samples were incubated at room temperature on a rocking plate for 20 min. The eluate was transferred to a 50 mL centrifuge tube and 1 mL of eluate was used for extraction using 100 µL of magnetic beads.

For RNA extraction, samples were incubated with magnetic beads for 30 min on a rotary shaker. The beads were washed with PBS three times and resuspended in 50 µL ultrapure water, heated at 100 °C for 10 min and then quickly chilled on ice. RNA was stored at -80 °C.

### ddRT-PCR

FCV, MNV or HAV RNA recovered by the ISO 15216-1 method and the bead assays were quantified using the One-Step dd-RT-PCR Advanced Kit for Probes (Bio-Rad Laboratories, Ltd.) according to manufacturer’s instructions and as described previously (Mykytczuk et al. 2017; Petronella et al. 2018). Primers and probes used to quantify FCV, MNV and HAV are listed in Table 1.

**Table 1:** List of primers and probes used in this study

Name	Sequence (5′–3′)	References
FCV-Q-A	GACACCTCCGACGAGTTATGC	Mattison et al. (2009)
FCV-Q-1	CCGGGTGGGACTGAGTGG	Mattison et al. (2009)
FCVFAM (probe)	CGCCTTACG/ZEN/GATATGAGCAGCCACATTAAC	Mattison et al. (2009)
MNVKS1	AGGTCATGCGAGATCAGCTT	Bae and Schwab (2008)
MNVKS2	CCAAGCTCTCACAAGCCTTC	Bae and Schwab (2008)
MNVSK3 (probe)	CAGTCTGCG/ZEN/ACGCCATTGAGAA	Bae and Schwab (2008)
SH-POLY-F	GAR TTT ACT CAG TGT TCA ATG AAT GT	Guevremont et al. (2006)
SH-POLY-R	GGC ATA GCT GCA GGA AAA TT	Guevremont et al. (2006)
SH-POLY-Q (probe)	TCTCCAAAA/ZEN/CGCTTTTTAGAAAGAGTCC	Guevremont et al. (2006)

The probes were labeled with Black quencher and FAM reporter dye for FCV and MNV, and HEX reporter dye for HAV

The probes for the detection and quantification of the viruses in this study were made with the Iowa Black quencher (Integrated DNA technologies), as this quencher produced the least background. The FAM reporter dye was used for probes to detect FCV and MNV, and the HEX reporter dye was used for HAV. All probes were made with an internal ZEN quencher to reduce background noise (Integrated DNA Technologies).

The thermocycling conditions were: 50 °C for 60 min, 95 °C for 10 min, 40 cycles of 95 °C for 30 s (Ramp = 2 °C/s) with an annealing temperature of 55 °C for FCV, and 53 °C for MNV and HAV, for 1 min (Ramp = 2 °C/s), and 98 °C for 10 min. ddRT-PCR results were analyzed using the QX200™ Droplet Digital system (Bio-Rad Laboratories Ltd.).

### Inactivation by Advanced Oxidation Process (AOP)

Twenty-five gram samples of each low-moisture food were inoculated with 100 µL of 5 × 10^6^ PFU/mL of FCV, 8 × 10^7^ PFU/mL of MNV, or 1 × 10^5^ PFU/mL of HAV. In triplicate, each food was exposed to 0 s (untreated), 30 s and 60 s of UV and hydrogen peroxide treatment. UV, ozone, and hydrogen peroxide treatment was applied to the samples using an AOP device (Clean Works©, Beamsville, ON Canada), which is an instrument consisting of a conveyor belt that moves through a treatment chamber with spray nozzles that release hydrogen peroxide (6%) at a flow rate of 50 mL/min, 4 UV-C bulbs (254 *λ*, 82 *ϕ*/watts each), and 4 ozone generating bulbs (187 *λ*, 2.3 mg/h ozone each). Therefore, for 30 s, and 60 s treatments, each sample came to contact with 830 µL and 1.67 mL of 6% v/v of hydrogen peroxide, 16.5 mg and 33 mg of ozone and 1.9 J/cm^2^ and 3.8 J/cm^2^ of UV irradiation, respectively. A heating fan functioned to distribute the hydrogen peroxide, in addition to maintaining the temperature around 48 °C.

For 30 s of treatment, the samples (pistachios and cornflakes in petri dishes, and chocolate in 6-well plates) were placed in a metal container on the conveyor belt and passed though the treatment chamber for 30 s (the manufacturer’s recommendation). For 60 s of treatment, the samples were placed in metal containers on the conveyor belt and held for an additional 30 s by attaching masking tape to the metal dish. Viruses were recovered from inoculated LMFs using the ISO-15216-1 method. Quantification of viral RNA was done by ddRT-PCR and the number infectious viruses was quantified by plaque assay as described above.

### Statistical Analysis

Statistical analysis was performed using Microsoft Excel 2016 to determine significant differences in viral inactivation between AOP treatment duration using the paired *t*-test.

## Results

### Virus Survival in Low-Moisture Foods

**Fig. 1:**
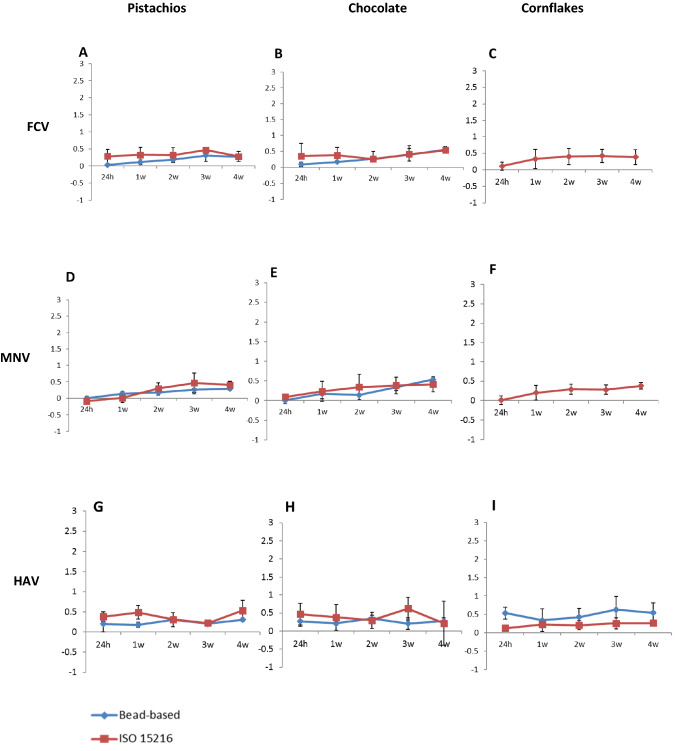
Log reduction of viral genome copies compared to *T*_0_. FCV A-C, MNV D-F, and HAV G-I recovered by the ISO-15216 method and the bead-based method in inoculated pistachios, chocolate, and cornflakes over 4-week incubation determined by ddRT-PCR. The data represent the mean of six replicates. The *Y* axis is log reduction compared to *T*_0_, the *X* axis is the time post-inoculation. Error bars represent standard deviation

We examined the survival of FCV, MNV and HAV in LMF by inoculation of pistachios, chocolate, cereals, and storage at room temperature (*RT*) for four weeks. The survival of the viruses on chocolate and pistachios was determined using both the ISO-15216 method and the bead-based method except for cereals (cornflakes) for which the bead-based assay had previously failed to recover FCV and MNV (Nasheri et al. 2020). Quantification of the recovered viral genomes was performed at different time points using ddRT-PCR and the results are presented as log reduction compared to *T*_0_. As shown in Fig. 1, the reduction in viral load was less than 0.5 log during the 4-week incubation for all the studied viruses and the LMF commodities. This finding indicates that the genomes of FCV, MNV, and HAV remain stable on LMF during long-term incubation. Moreover, despite the premise that the employment of bead-based assays leads to extraction of “intact” viral capsids and therefore provide a better insight into the presence of infectious viral particles in food commodities, the results of the bead-based assay and the ISO-15216 methods demonstrate similar decline rate in viral genome copy number during the 4-week storage (Fig. 1).

**Fig. 2:**
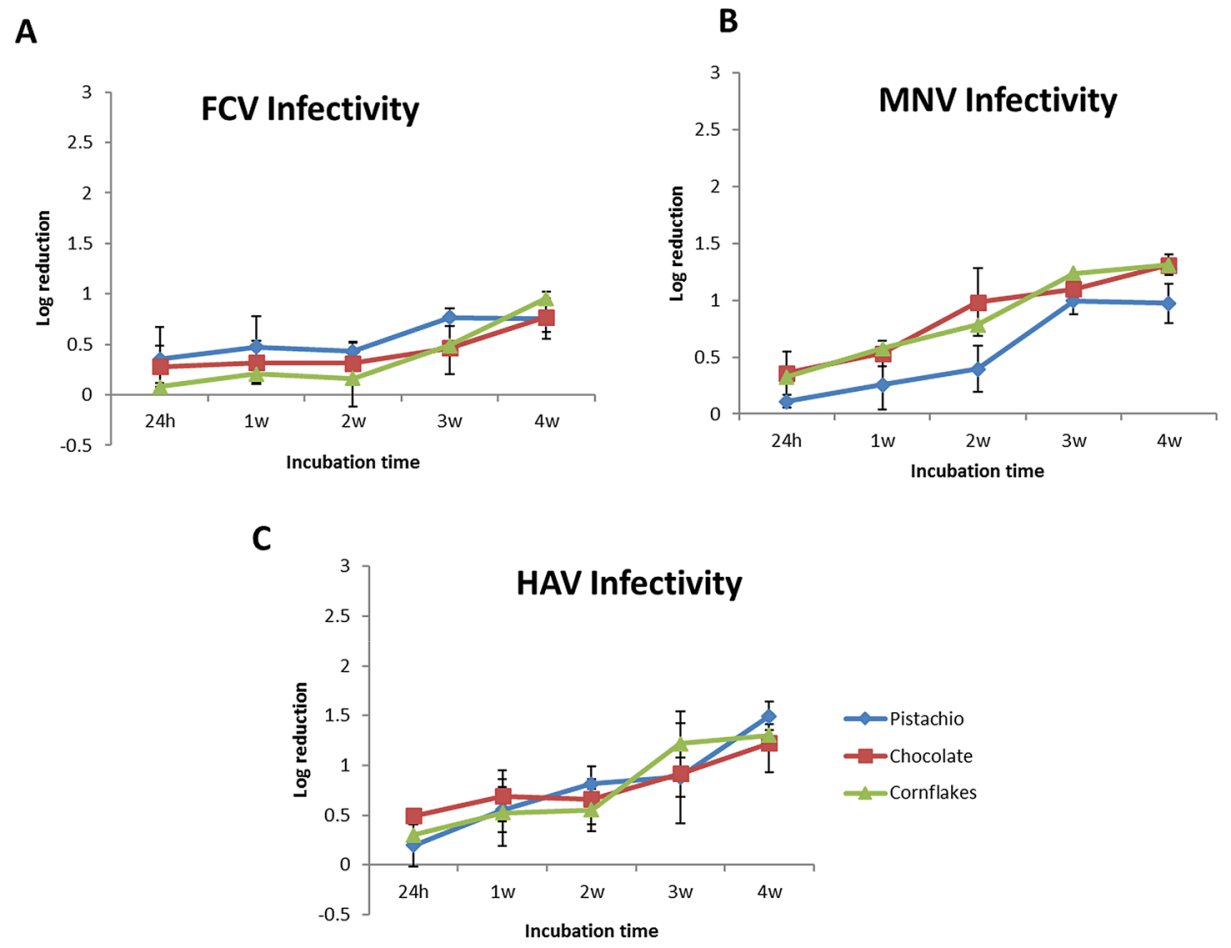
Log reduction in infectious viral particles for **a** FCV, **b** MNV, and **c** HAV over 4-week incubation determined by plaque assay. The ISO-15216 method was employed for virus recovery. The data represent the mean of six replicates. The error bars represent standard deviation

To further examine the viral survival in LMFs, we performed infectivity assay (plaque assay) on viruses that were isolated at different time points post-inoculation and compared the infectivity results to *T*_0_. Since the viruses captured by the bead-based method could not be used for plaque assay, only the viruses isolated by the ISO-15216 were subjected to plaque assay. As shown in Fig. 2, while the infectivity of FCV decreases gradually in all studied LMFs during the 4-week incubation time to less than 1 log (0.83 ± 0.1 log) compared to *T*_0_ (Fig. 2a), the overall infectivity of MNV and HAV reduced to 1.20 ± 0.21 log and 1.34 ± 0.14 log (Fig. 2b, c), respectively. These data are in line with previous reports that foodborne viruses are persistent in foods and the environment over a long period of time (Cook et al. 2018, 2016; Fallahi and Mattison 2011).

### Inactivation by Advanced Oxidative Process

**Fig. 3:**
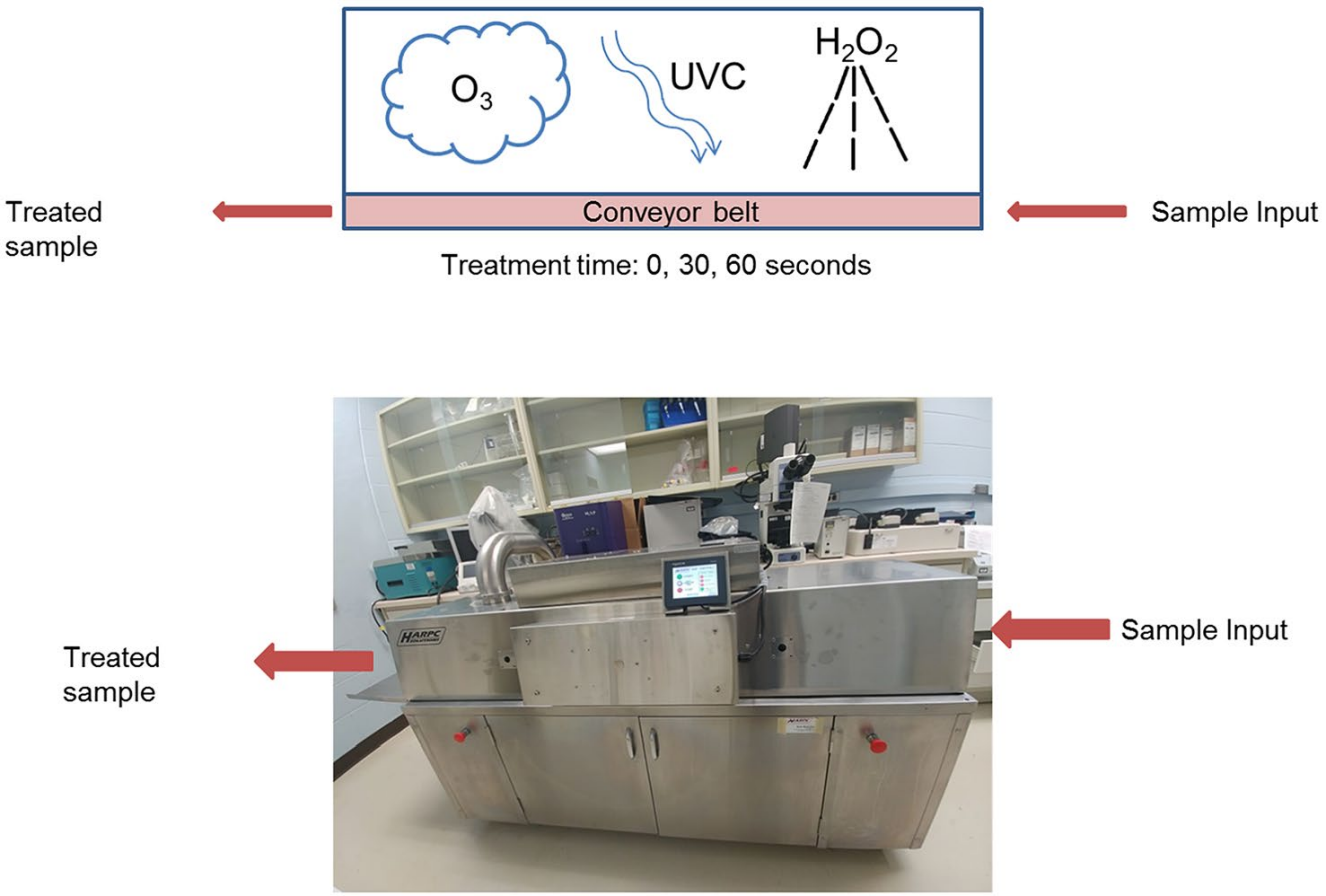
Schematic presentation of the processes used in the Advanced Oxidative Process (AOP) unit, which is an instrument consisting of a conveyor belt that moves through a treatment chamber with spray nozzles that release hydrogen peroxide (6%) at a flow rate of 50 mL/min, 4 UV-C bulbs (254 *λ*, 82 *ϕ*/watts each), and 4 ozone generating bulbs (187 λ, 2.3 mg/h ozone each)

In this study, we evaluated the efficacy of using the Advanced Oxidative Process (AOP) technology as a new method to inactivate foodborne viral pathogens in LMFs. The AOP unit has 4 components; UV-C light, ozone, heat, and hydrogen peroxide vapor. The unit consisted of a UV light box that had a combination of UV-C (254 nm) and ozone generating (185 nm) lamps. Hydrogen peroxide was introduced via a vaporizer at 6% concentration. A heating fan functioned to distribute the hydrogen peroxide, in addition to maintaining the temperature around 48 °C (Fig. 3).

**Fig. 4:**
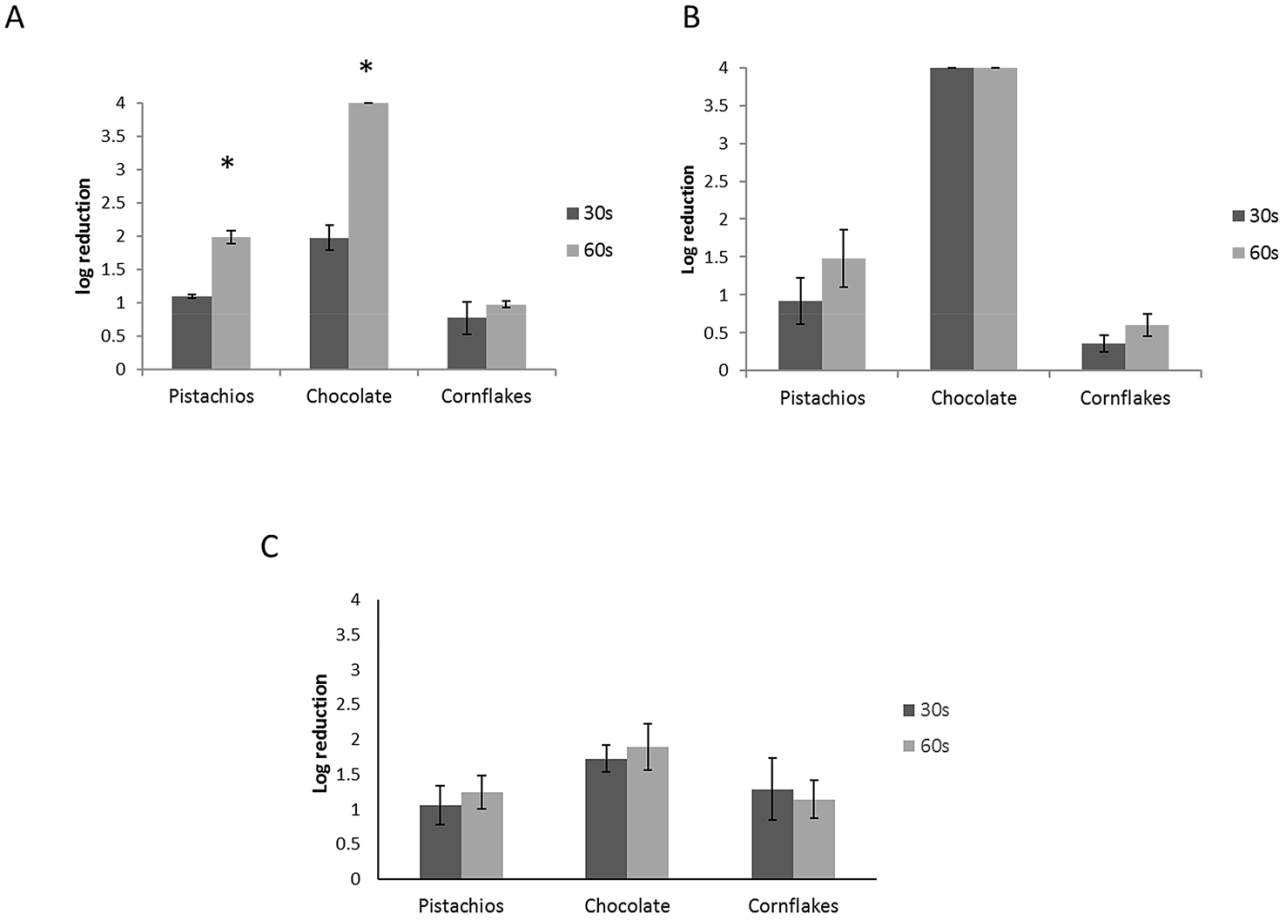
The average log reduction in viral infectivity after treatment in AOP for 30 and 60 s for **a** FCV, **b** MNV, and **c** HAV compared to the untreated controls in all three LMFs. The data represent the mean of three replicates. The error bars represent standard deviation. *P < 0.01

Artificially inoculated pistachios, chocolate, and cornflakes with FCV, MNV and HAV were placed on a petri dish and kept in the AOP unit for 30 or 60 s. We did not observe any change in the appearance of the treated LMFs. Viral inactivation was examined by comparing the viral titre of the recovered viruses from treated samples to the inoculated but untreated samples using both plaque assay and ddRT-PCR (Fig. 4 and Supplementary Fig. 1).

As shown in Fig. 4a, FCV inactivation, examined by plaque assay, was close to 2 log for 30 s and 4 log (complete) for 60 s treatment of chocolate, which has a flat surface. The reduction in FCV titre was 1.1 log and 2 log for 30 s and 60 s treatments in pistachios, respectively, while in cornflakes it was less than 1 log (0.77 log and 0.97 log for 30 s and 60 s treatments, respectively). Also, the data suggest that the increase in the treatment time from 30 to 60 s did not significantly enhance viral inactivation in cornflakes (*P* > 0.05), while the increase in treatment time significantly enhanced viral inactivation on pistachios and chocolate (*P* < 0.01). However, the analysis of viral inactivation by examining the survival of the FCV genome by ddRT-PCR in Supplementary Fig. 1 reveals decreased inactivation compared to the infectivity assay (Supplementary Fig. 1A). This indicates that a proportion of surviving genomes that are quantified by ddRT-PCR do not belong to infectious particles.

Similar observations were made regarding the MNV inactivation (Fig. 4b); no infectious virus was recovered from the treated chocolate (30 s and 60 s), indicating that viral inactivation was complete (4 log) while the inactivation in pistachios was 0.9 log and 1.5 log for 30 s and 60 s treatments, respectively. In cornflakes, MNV inactivation was only 0.4 log and 0.6 log for 30 s and 60 s treatments, respectively. The increase in the treatment time from 30 to 60 s did not significantly enhance MNV inactivation in pistachios and cornflakes (*P* > 0.05). Like FCV, the MNV genome was more resistant to inactivation by AOP (Supplementary Fig. 1b) and demonstrated reduced inactivation compared to MNV infectivity assay (Fig. 4b), suggesting that all recovered genomes might not be infectious.

Contrary to FCV and MNV, HAV inactivation in chocolate was never complete and was less than 2 log (1.7 log for 30 s and 1.9 log for 60 s treatments). In pistachios, HAV inactivation was 1.1 log and 1.25 log and in cornflakes, it was 1.3 and 1.15 log for 30 s and 60 s treatments, respectively. The increase in the treatment time did not change the inactivation outcome significantly in any of the studied LMFs. Similar to observations made for FCV and MNV, the genome of HAV is more persistent, compared to its infectivity, following AOP treatment (Supplementary Fig. 1c). In addition, the duration of treatment did not have a significant effect on inactivation (*P* > 0.05). Moreover, the ddRT-PCR data suggest that the genome of HAV is more resistant to elimination by the AOP treatment compared to FCV and MNV, as the reduction in the RNA genome never exceeded 0.65 log (Supplementary Fig. 1), while at least 2 log reduction was observed for the genome of FCV and MNV in chocolate after treatment for 60 s. Altogether, these data suggest that the virucidal effect of AOP treatment depends on the matrix and the surface of the treated LMF.

## Discussion

We have formerly compared the efficacy of the ISO 15216:2017 method with the magnetic beads assays for the isolation of HAV, MNV and FCV in shelled pistachios, chocolate liqueur and cereals and demonstrated that higher recovery efficiencies can be obtained by the bead-based assays compared with the ISO-15216 method (Nasheri et al. 2020). However, the bead-based method failed to recover FCV and MNV from cornflakes and for this reason, the bead-based assay was not employed to examine FCV and MNV persistence in cornflakes (Nasheri et al. 2020). One potential explanation for this observation is that the high carbohydrate content of cornflakes outcompeted the PGM-coated beads in recovering FCV and MNV, as it has been demonstrated that HBGA/sialic acid-like components in certain produce can also compete with PGM binding (Suresh et al. 2019; Esseili et al. 2012). This can be further confirmed by the fact that anti-HAV coated beads, that do not rely on carbohydrate interaction for capturing HAV, could efficiently recover this virus from cornflakes (Fig. 1i).

Another advantage of the ISO-15216 method over the bead-based assay is that it allows for performing infectivity assay on the precipitated viruses. To date, we have not found an efficient method for the elution of the captured viruses by magnetic beads without having a drastic negative impact on their infectivity. Therefore, the viruses that were captured by the bead-based method were not subjected to plaque assay.

The survival data revealed that FCV is slightly more persistent in LMF compared to MNV and HAV, as the overall reduction in infectivity at W4 was less than 1 log for FCV, while the loss in infectivity of MNV and HAV was around 1.2 log and 1.34 log, respectively. Our findings are in accordance with the previous reports about survival of foodborne viruses on food-contact surfaces and fomites that the reduction in viral infectivity is linear overtime, with a less than 1.5 log after 4 weeks incubation (Cook et al. 2016).

Except for cornflakes, the persistence of the viral genome was examined using two different extraction methods; the bead-based method and the ISO 15216 method. Both methods generated similar results with a reduction of approximately 0.5 log for all three viruses, with a similar pattern of viral genome reduction rate over the incubation time in all tested LMFs (Fig. 1). The reduction of the viral genome is significantly lower than the decrease in viral infectivity, which suggests that the presence of the viral genome does not necessarily correlate with the presence of infectious particles. Thus, the bead-based assay might not be a true indicator of viral infectivity. Altogether, these data demonstrate that these viruses persist in an infective state, regardless of the food matrix, for a relatively long period of time in LMFs and support the previous finding that low humidity might even be beneficial for viral persistence (Colas de la Noue et al. 2014).

It has been estimated that a class I recall of LMFs due to pathogen contamination leads to a median loss in corporate value of $243,000,000 in the USA (Gomez and Marks 2020). To control foodborne pathogens in LMFs, a combination of pre-and post-harvest sanitation, Good Manufacturing Practice (GMP), and decontamination interventions are required (Scott et al. 2009). Strategies that could reduce the risk of pathogen contamination not only reduce the healthcare and societal costs associated with foodborne illnesses, but also have a positive economic impact on the industry. Thus, novel and effective pathogen control technologies need to be developed, tested and implemented for improved food safety of LMFs. For this reason, we collaborated with industry to examine the efficacy of the AOP system against foodborne viral pathogens.

Although the effect of UV-C, ozone, and hydrogen peroxide for viral inactivation has been evaluated individually, the combined virucidal efficacy of these strategies has never been examined. The AOP system enabled us to employ all these strategies in a single unit (Fig. 3). We observed while the AOP treatment led to 100% (4 log) inactivation of FCV and MNV in chocolate (Fig. 4), the treatment of inoculated pistachios and cornflakes caused less than 90% (1 log) reduction in viral infectivity. This finding indicates that either the food matrix or the surface of treatment can influence the outcome of inactivation. The effect of surface on viral inactivation has been shown previously for hydrogen peroxide vapor (Becker et al. 2019a), ozone (Hudson et al. 2007), and UV-C (Butot et al. 2018) individually. The greater inactivation of viruses on smooth surfaces has been attributed to the protective effect of shadowing in rough surfaces (Butot et al. 2018). Thus, the smooth surface of chocolate could potentially provide less of a protective shadow compared to the surfaces of pistachios and cornflakes, which could explain the greater inactivation outcome on chocolate than pistachios and cornflakes. Also, it has been demonstrated that the presence of organic matter diminishes the ozone treatment efficacy (Hudson et al. 2007). We speculate that the successful application of AOP relies on the treatment components, i.e., UV-C, ozone and hydrogen peroxide vapor reaching all the virus particles directly and if the viruses are present in cracks, crevices or openings in the food and surfaces, the viruses may be protected and thus survive. Our data are also consistent with the previous finding that the duration of treatment, as long as it is over 20 s, does not make a significant difference in the inactivation outcome (Butot et al. 2018).

The impact of AOP treatment has recently been explored for inactivation of foodborne bacteria on LMFs (Hasani et al. 2020). It has been demonstrated that the efficacy of inactivation of *Salmonella* by AOP treatment on LMFs could be matrix-dependent as inactivation on chocolate and cornflakes is approximately 6 log, while it is less than 4 log on pistachios, and similar observations were made for inactivation of *L. monocytogenes* (Hasani et al. 2020). AOP treatment of *L. monocytogenes* on the surface of apples for 30 s to 120 s, led to approximately 3 log reduction, and similar to our observations, the increase in treatment time did not significantly improve the inactivation outcome (Murray et al. 2018).

Similar to other studies, we have observed that the detection and quantification of viral RNA, does not necessarily correlate with viral infectivity (Cook et al. 2018, 2016; Yeargin et al. 2016). In survival assays and AOP experiments, viral RNA was detected at higher titre compared to viral infectivity.

Herein, we did not determine the final concentration of ozone and hydrogen peroxide on the treated surfaces, nor did we alter the pre-set settings of the AOP unit. Further AOP research works needs to be performed on a combination of food and surfaces as well as different intensities for each component of the AOP unit to determine whether the inactivation outcome could be improved. Moreover, the efficacy of AOP on pathogen removal from high-risk raw LMFs, such as dried fruits and berries needs to be explored in future.

In conclusion, we employed the bead-based method and the ISO 15216 method to demonstrate that foodborne viruses are persistent for a long period in LMFs. Furthermore, we demonstrated that depending on the food matrix and its surface, the AOP treatment could be an effective means to improve the virological food safety of LMFs.

## Supplementary Information

Below is the link to the electronic supplementary material.

Supplementary file1 (DOCX 49 KB)

## References

[CR1] Anonymous. (2019). Retrieved from http://www.inspection.gc.ca/about-the-cfia/newsroom/food-recall-warnings/complete-listing/2016-03-10/eng/1457627335799/1457627341228.

[CR2] Bae J, Schwab KJ (2008). Evaluation of murine norovirus, feline calicivirus, poliovirus, and MS2 as surrogates for human norovirus in a model of viral persistence in surface water and groundwater. Applied and Environment Microbiology.

[CR3] Becker B, Dabisch-Ruthe M, Pfannebecker J (2019). Inactivation of murine norovirus on fruit and vegetable surfaces by vapor phase hydrogen peroxide. Journal of Food Protection.

[CR4] Bidawid S, Malik N, Adegbunrin O, Sattar SA, Farber JM (2003). A feline kidney cell line-based plaque assay for feline calicivirus, a surrogate for Norwalk virus. Journal of Virological Methods.

[CR5] Bosch A, Gkogka E, Le Guyader FS, Loisy-Hamon F, Lee A, van Lieshout L, Marthi B, Myrmel M, Sansom A, Schultz AC, Winkler A, Zuber S, Phister T (2018). Foodborne viruses: Detection, risk assessment, and control options in food processing. International Journal of Food Microbiology.

[CR6] Brie A, Boudaud N, Mssihid A, Loutreul J, Bertrand I, Gantzer C (2018). Inactivation of murine norovirus and hepatitis A virus on fresh raspberries by gaseous ozone treatment. Food Microbiology.

[CR7] Butot S, Cantergiani F, Moser M, Jean J, Lima A, Michot L, Putallaz T, Stroheker T, Zuber S (2018). UV-C inactivation of foodborne bacterial and viral pathogens and surrogates on fresh and frozen berries. International Journal of Food Microbiology.

[CR8] Carvalho C, Thomas H, Balogun K, Tedder R, Pebody R, Ramsay M, Ngui S (2012). A possible outbreak of hepatitis A associated with semi-dried tomatoes, England, July–November 2011. Eurosurveillance.

[CR9] Colas de la Noue A, Estienney M, Aho S, Perrier-Cornet JM, de Rougemont A, Pothier P, Gervais P, Belliot G (2014). Absolute humidity influences the seasonal persistence and infectivity of human norovirus. Applied and Environment Microbiology.

[CR10] Cook N, Bertrand I, Gantzer C, Pinto RM, Bosch A (2018). Persistence of hepatitis A virus in fresh produce and production environments, and the effect of disinfection procedures: A review. Food and Environmental Virology.

[CR11] Cook N, Knight A, Richards GP (2016). Persistence and elimination of human norovirus in food and on food contact surfaces: A critical review. Journal of Food Protection.

[CR12] Cox C, Cao S, Lu Y (2009). Enhanced detection and study of murine norovirus-1 using a more efficient microglial cell line. Virology Journal.

[CR13] Dancho BA, Chen H, Kingsley DH (2012). Discrimination between infectious and non-infectious human norovirus using porcine gastric mucin. International Journal of Food Microbiology.

[CR14] Donnan EJ, Fielding JE, Gregory JE, Lalor K, Rowe S, Goldsmith P, Antoniou M, Fullerton KE, Knope K, Copland JG, Bowden DS, Tracy SL, Hogg GG, Tan A, Adamopoulos J, Gaston J, Vally H (2012). A multistate outbreak of hepatitis A associated with semidried tomatoes in Australia, 2009. Clinical Infectious Diseases.

[CR15] Esseili MA, Wang Q, Saif LJ (2012). Binding of human GII.4 norovirus virus-like particles to carbohydrates of romaine lettuce leaf cell wall materials. Applied and Environment Microbiology.

[CR16] Fallahi S, Mattison K (2011). Evaluation of murine norovirus persistence in environments relevant to food production and processing. Journal of Food Protection.

[CR17] Gomez CB, Marks BP (2020). Monetizing the impact of food safety recalls on the low-moisture food industry. Journal of Food Protection.

[CR18] Guevremont E, Brassard J, Houde A, Simard C, Trottier YL (2006). Development of an extraction and concentration procedure and comparison of RT-PCR primer systems for the detection of hepatitis A virus and norovirus GII in green onions. Journal of Virological Methods.

[CR19] Hasani M, Wu F, Hu K, Farber J, Warriner K (2020). Inactivation of Salmonella and Listeria monocytogenes on dried fruit, pistachio nuts, cornflakes and chocolate crumb using a peracetic acid-ethanol based sanitizer or Advanced Oxidation Process. International Journal of Food Microbiology.

[CR20] Hudson JB, Sharma M, Petric M (2007). Inactivation of Norovirus by ozone gas in conditions relevant to healthcare. Journal of Hospital Infection.

[CR21] ISO (2017). Microbiology of the food chain – horizontal method for determination of hepatitis A virus and norovirus using real-time RT-PCR – part 1: method for quantification. ISO 15216–1:2017.

[CR22] Ly V, Parreira VR, Sanchez-Maldonado AF, Farber JM (2020). Survival and virulence of listeria monocytogenes during storage on chocolate liquor, corn flakes, and dry-roasted shelled pistachios at 4 and 23 °C. Journal of Food Protection.

[CR23] Mattison K, Brassard J, Gagne MJ, Ward P, Houde A, Lessard L, Simard C, Shukla A, Pagotto F, Jones TH, Trottier YL (2009). The feline calicivirus as a sample process control for the detection of food and waterborne RNA viruses. International Journal of Food Microbiology.

[CR24] Mbithi JN, Springthorpe VS, Sattar SA (1991). Effect of relative humidity and air temperature on survival of hepatitis A virus on environmental surfaces. Applied and Environment Microbiology.

[CR25] Murray K, Moyer P, Wu F, Goyette JB, Warriner K (2018). Inactivation of listeria monocytogenes on and within apples destined for caramel apple production by using sequential forced air ozone gas followed by a continuous advanced oxidative process treatment. Journal of Food Protection.

[CR26] Mykytczuk O, Harlow J, Bidawid S, Corneau N, Nasheri N (2017). Prevalence and molecular characterization of the hepatitis E virus in retail pork products marketed in Canada. Food Environment Virology.

[CR27] Nasheri N, Harlow J, Chen A, Corneau N, Bidawid S (2020). Evaluation of bead-based assays in the isolation of foodborne viruses from low-moisture foods. Journal of Food Protection.

[CR28] Nasheri N, Vester A, Petronella N (2019). Foodborne viral outbreaks associated with frozen produce. Epidemiology and Infection.

[CR29] Park JH, Jeong HS, Lee JS, Lee SW, Choi YH, Choi SJ, Joo IS, Kim YR, Park YK, Youn SK (2015). First norovirus outbreaks associated with consumption of green seaweed (*Enteromorpha* spp.) in South Korea. Epidemiology and Infection.

[CR30] Petronella N, Ronholm J, Suresh M, Harlow J, Mykytczuk O, Corneau N, Bidawid S, Nasheri N (2018). Genetic characterization of norovirus GII.4 variants circulating in Canada using a metagenomic technique. BMC Infectious Diseases.

[CR31] Rzezutka A, Cook N (2004). Survival of human enteric viruses in the environment and food. FEMS Microbiology Reviews.

[CR32] Sakon N, Sadamasu K, Shinkai T, Hamajima Y, Yoshitomi H, Matsushima Y, Takada R, Terasoma F, Nakamura A, Komano J, Nagasawa K, Shimizu H, Katayama K, Kimura H (2018). Foodborne outbreaks caused by human norovirus GII.P17-GII.17-contaminated Nori, Japan, 2017. Emerging Infectious Diseases.

[CR33] Sanchez-Maldonado AF, Lee A, Farber JM (2018). Methods for the control of foodborne pathogens in low-moisture foods. Annual Review of Food Science and Technology.

[CR34] Santillana Farakos SM, Schaffner DW, Frank JF (2014). Predicting survival of Salmonella in low-water activity foods: An analysis of literature data. Journal of Food Protection.

[CR35] Scott VN, Chen Y, Freier TA, Kuehm J, Moorman M, Meyer J, Morille-Hinds T, Post L, Smoot L, Hood S, Shebuski J, Banks J (2009). Control of *Salmonella* in low-moisture foods I: Minimizing Entry of *Salmonella* into a processing facility. Food Protection Trends.

[CR36] Shukla S, Cho H, Kwon OJ, Chung SH, Kim M (2018). Prevalence and evaluation strategies for viral contamination in food products: Risk to human health-a review. Critical Reviews in Food Science and Nutrition.

[CR37] Suresh M, Harlow J, Nasheri N (2019). Evaluation of porcine gastric mucin assay for detection and quantification of human norovirus in fresh herbs and leafy vegetables. Food Microbiology.

[CR38] Yeargin T, Buckley D, Fraser A, Jiang X (2016). The survival and inactivation of enteric viruses on soft surfaces: A systematic review of the literature. American Journal of Infection Control.

[CR39] Yeargin T, Gibson KE (2018). Key characteristics of foods with an elevated risk for viral enteropathogen contamination. Journal of Applied Microbiology.

[CR40] Yu JH, Kim NY, Koh YJ, Lee HJ (2010). Epidemiology of foodborne Norovirus outbreak in Incheon, Korea. Journal of Korean Medical Science.

[CR41] Zonta W, Mauroy A, Farnir F, Thiry E (2016). Virucidal efficacy of a hydrogen peroxide nebulization against murine norovirus and feline calicivirus, two surrogates of human norovirus. Food and Environmental Virology.

