# Supplementary material for: Survival and Inactivation by Advanced Oxidative Process of Foodborne Viruses in Model Low-Moisture Foods
Source: Food Environ Virol. 2021 Jan 27;13(1):107–16. doi: 10.1007/s12560-020-09457-7 (PMC7882587; doi:10.1007/s12560-020-09457-7)
Supplement: Supplementary file 1 — Supplementary file1 (DOCX 49 KB) [file 12560_2020_9457_MOESM1_ESM.docx]

**Supplementary Table 1.** Average recovery rates (RR) in percentage obtained from each LMF using the ISO 15216 method. RR was calculated by comparison of the absolute genome copy number of the virus recovered from the LMFs at T_0_ to the absolute genome copy number of the virus aliquot used to inoculate the LMFs

|  | HAV | FCV | MNV |
| --- | --- | --- | --- |
| Cornflakes | 8.55±3.1 | 7.05±2.7 | 6.6±1.2 |
| Chocolate | 7.9±2.5 | 2.3±0.7 | 1.9±0.8 |
| Pistachios | 2.8±1.6 | 1.8±0.8 | 1.6±0.7 |

**Supplementary Table 2.** Average retrieved titre (PFU/mL) at T_0,_ using the ISO 15216, for each virus and LMF is demonstrated. Average recovery rates in percentage obtained from each LMF after infectivity assay is also shown.

|  | Retrieved Titre at T0 | | | Recovery rate (%) | | |
| --- | --- | --- | --- | --- | --- | --- |
| Matrix | HAV | FCV | MNV | HAV | FCV | MNV |
| Cornflakes | 1.32E+03 | 7.78E+03 | 1.38E+03 | 2.9 | 0.55 | 0.6 |
| Chocolate | 2.88E+03 | 2.65E+04 | 1.07E+04 | 6.8 | 0.8 | 1.2 |
| Pistachios | 2.68E+04 | 3.78E+04 | 4.67E+03 | 1.3 | 0.2 | 0.3 |

**Supplementary Figure 1.** Average log reduction in genomic RNA after treatment with AOP. Error bars represent standard deviation.

**
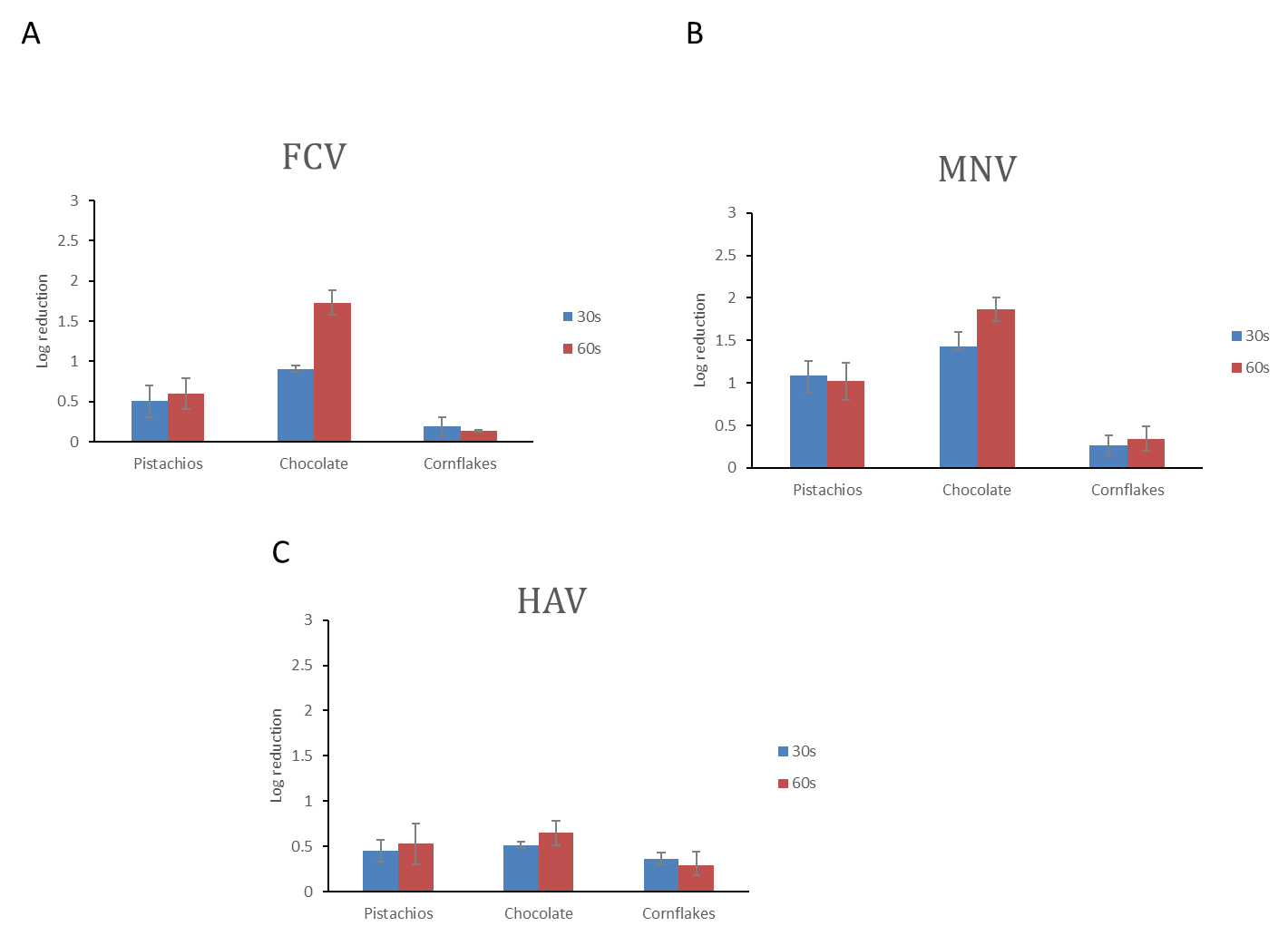
**
